# Avoiding the Trap of Misdiagnosis: Valuable Teaching Points Derived from a Case of Longstanding Popliteal Artery Entrapment Syndrome

**DOI:** 10.1155/2018/3214561

**Published:** 2018-02-14

**Authors:** Heitham Wady, Zain Badar, Zerwa Farooq, Palma Shaw, Katsuhiro Kobayashi

**Affiliations:** SUNY Upstate Medical Center, Syracuse, NY, USA

## Abstract

Popliteal artery entrapment syndrome (PAES), a condition predominantly affecting young individuals, is a rare clinical entity that can result in significant morbidity. The presence of lower limb pain and claudication in young, physically active individuals should prompt consideration for PAES. Early diagnosis and management is crucial to prevent long-term complications; however, diagnosis is fraught with challenges due to the rarity of the disease and its similar clinical presentation with more common conditions. We present a case of a young female with PAES who was misdiagnosed and underwent a tarsal tunnel release for suspected tarsal tunnel syndrome and subsequent fasciotomies for presumed chronic exertional compartment syndrome (CECS) without any relief. We outline the insidious undiagnosed course of her condition over a period of 12 years, discuss teaching points of how to recognize key differences of PAES and associated conditions, and provide recommendations for how to make the right diagnosis.

## 1. Introduction

Popliteal artery entrapment syndrome (PAES), a consequence of aberrant positioning of structures surrounding the popliteal artery within the popliteal fossa, may present due to a variety of anatomical differences. The classification scheme is summarized in Table 1.

Classically, the condition affects young men and those individuals who are highly physically active, including military personnel and long-distance runners [1, 2]. Prolonged exercise causing hypertrophy of the gastrocnemius muscle is commonly the mechanism behind the accelerated development of symptoms in these patients [1, 2]. PAES can also present in a congenital form, occurring secondary to errors in embryological development of the extremities leading to gross anatomical abnormalities [3]. In the context of these demographics, consideration of PAES in the female population can be overlooked. We present a case of a young female with PAES and its insidious undiagnosed course over a period of 12 years.

## 2. Case Report

A 30-year-old female presented following a fall during a workout session, when her leg “gave out.” As an avid runner, she noted progressive worsening of her daily activities and exercise intolerance due to pain, numbness, and a burning sensation in her right lower extremity, symptoms that had been present for 12 years. She worked as a physical therapist and while assessing her pulse a decreased ankle-brachial index was noted. She had a history of a tarsal tunnel release 14 years ago for presumed tarsal tunnel syndrome followed by a four-compartment fasciotomy for supposed chronic exertional compartment syndrome (CECS) 10 years ago.

Physical exam of the right leg demonstrated a normal femoral pulse, while the popliteal pulse was absent with diminished posterior tibial and dorsalis pedis pulses. Tenderness to palpation was elicited overlying the right tibia.

**Figure 1:**
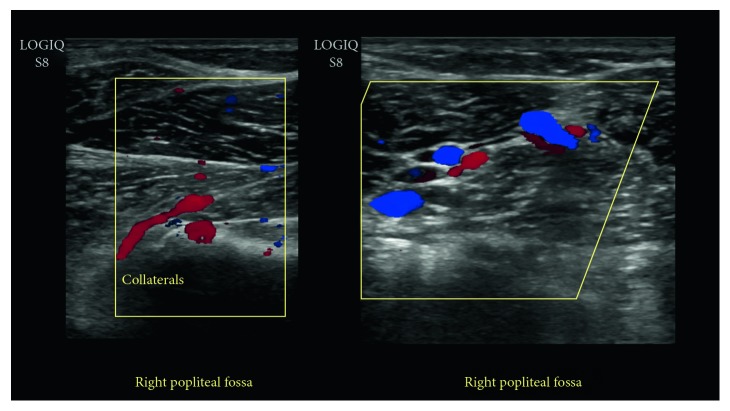
Duplex ultrasound of the right popliteal fossa demonstrating numerous collaterals. Native popliteal artery not clearly identified.

**Figure 2:**
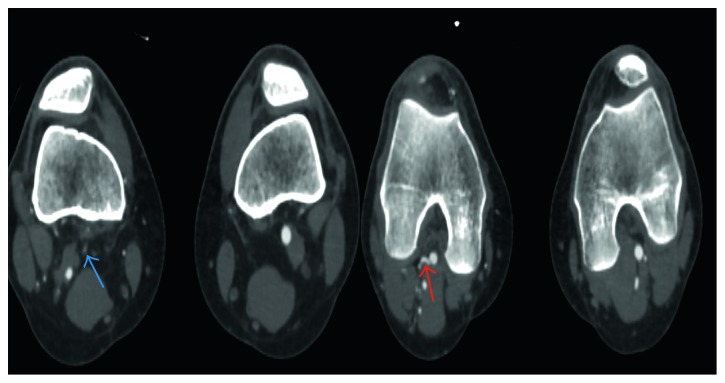
CTA demonstrating complete occlusion of the proximal right popliteal artery (blue arrow) with reconstitution of flow distally from robust collateral vessels (red arrow).

**Figure 3:**
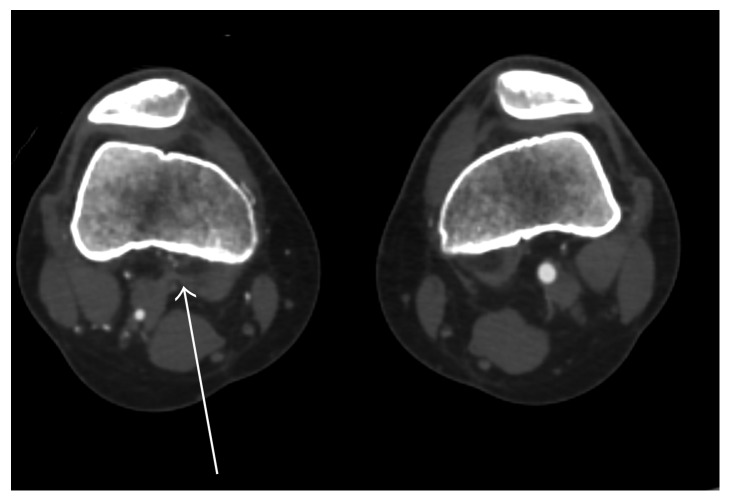
A band of soft tissue density (white arrow) is seen anterior to the right popliteal artery just distal to the occlusion.

**Figure 4:**
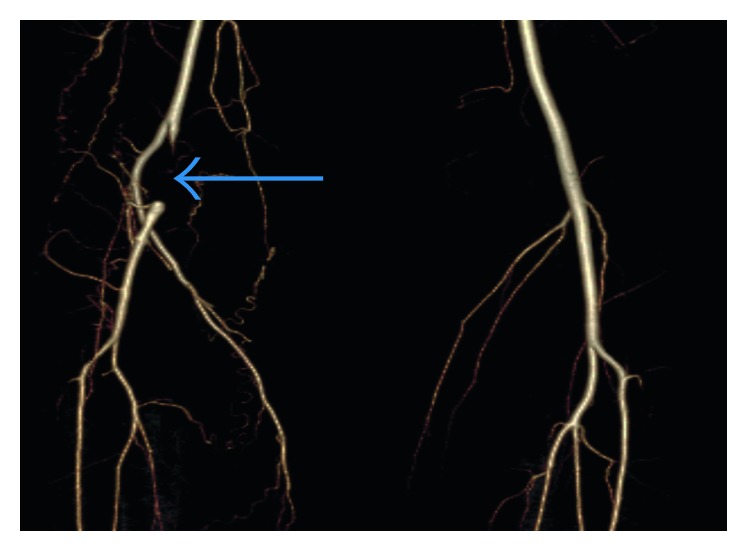
3D volume reconstruction obtained from axial source CTA images demonstrating occlusion of the right popliteal artery (blue arrow) with multiple adjacent collaterals.

Radiographs showed no evidence of a fracture. A duplex ultrasound demonstrated normal triphasic waveforms extending from the right superficial femoral artery caudally to the proximal popliteal artery. Distally, the native popliteal artery was not clearly identified. Multiple collaterals within the popliteal fossa were visualized (Figure 1). CT angiography (CTA) of the lower extremities showed complete occlusion of the proximal right popliteal artery with significant reconstitution of flow distally from robust collateral vessels (Figure 2). A band of soft tissue density was visualized anterior to the right popliteal artery just distal to the occlusion, suggestive of a fibrous band contributing to the occlusion (Figure 3). A 3D volume reconstruction obtained from axial source CTA images showed occlusion of the right popliteal artery with adjacent collaterals (Figure 4). The imaging and clinical findings correlated with PAES type IV.

**Figure 5:**
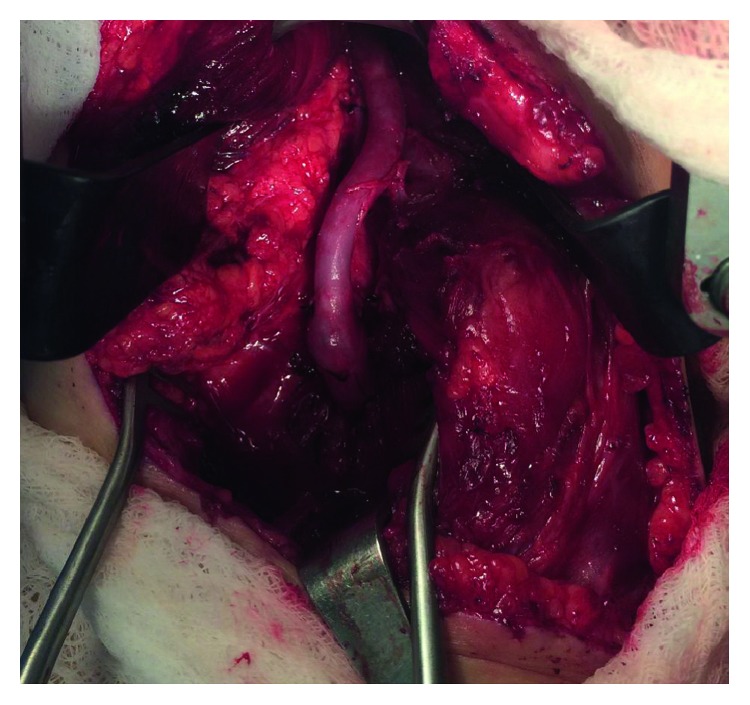
Intraoperative photo demonstrating successful interpositional bypass of the popliteal artery.

Based on these findings, the entrapment was not amenable to endovascular repair with minimally invasive techniques. Vascular surgery recommended right popliteal artery entrapment release coupled with popliteal-to-popliteal artery bypass and right lower extremity venous mapping. The two heads of the gastrocnemius muscles were split to expose the distal aspect of the popliteal artery. Small fibrous bands were removed off the popliteal artery followed by insertion of a bypass graft (Figure 5). Flow to the distal limb was excellent demonstrated by palpable pedal pulses on exam. Follow-up three weeks later showed palpable dorsalis pedis and posterior tibial pulses bilaterally, and a follow-up Duplex ultrasound of the right lower extremity revealed a patent bypass graft. The patient noted resolution of her original symptoms of lower extremity burning and numbness.

## 3. Discussion

Popliteal artery entrapment syndrome is a rare clinical entity affecting young individuals, causing symptoms of vascular compromise. Delay in diagnosis or misdiagnosis occurs commonly as patients lack vascular risk factors that would otherwise predispose them to symptoms associated with atherosclerosis and claudication [4]. Furthermore, PAES may often be subclinical in nature, and the prevalence of the condition may be deflated due to underreporting [5, 6]. As PAES predominantly affects young men [7–9], reaching a diagnosis in a female patient may prove to be more challenging, as demonstrated by this case, which remained undiagnosed for more than 12 years.

One of the challenges faced in diagnostic medicine is differentiating disease processes that overlap in clinical presentation. In the case of PAES, its clinical presentation and patient population closely mirror those of CECS; both conditions predominantly affect young, well-conditioned, physically active individuals who present with exercise-induced claudication or paresthesia [10–13]. However, key differences exist that may help distinguish the two: patients with PAES tend to be older than CECS patients, present with unilateral pain, are less likely to have elevated compartment pressures, and are far more likely to test positive during entrapment screening studies [10]. In the case of this patient, although she presented with symptoms at an earlier than expected age, measured compartment pressures were normal, the pain was unilateral, and four-compartment fasciotomy failed to provide resolution of symptoms. Thus, in such cases, PAES should be considered with further workup pursued.

Obtaining a good history of symptom onset and exacerbations along with a proper physical exam assessing lower extremity pulses can aid in diagnosis. Doppler ultrasonography is a useful inexpensive and noninvasive initial investigation to suggest the diagnosis [14, 15]. However, Doppler US without the use of provocative maneuvers can result in false negatives [11, 15]. Imaging in a relaxed position, as well as in resisted plantar flexion, or imaging after exercise once the symptoms are redemonstrated can improve sensitivity and decrease false negatives [15, 16]; however, this may result in false positives, so clinical correlation becomes extremely pertinent [17]. In the present case, manipulative maneuvers were not performed. The findings of prominent collaterals in the popliteal fossa in conjunction with poor visualization of the native popliteal artery on ultrasonography, along with the absence of the distal popliteal pulse, raised concerns for PAES.

Further testing can be done with CTA. MRI/MRA also helps to evaluate the vasculature, delineate the surrounding musculature, and provide an alternative for patients with contraindications to CTA [14–20]. This can assess the degree of pathology, assist in preoperative planning, and help to rule out conditions with similar clinical presentation such as cystic adventitial disease [14]. Some studies recommend provocative testing with MRI/MRA and CTA imaging in resisted plantar flexion to increase sensitivity, especially when functional PAES is suspected [16].

The insidious course of PAES may have serious long-term ramifications in an otherwise healthy individual. The abnormal anatomy of the popliteal fossa can produce continual compression of the popliteal artery, causing endothelial damage resulting in the formation of aneurysm, thrombosis, or embolism. Any of these sequelae in a patient can trigger ischemia and potentially threaten limb viability [4]. Early intervention is essential to adequate patient care. Once accurate diagnosis is established, long-term surgical outcome has shown to be excellent in reported patients [21].

Also in consideration is gender predilection of PAES towards males. Further investigative efforts should be geared towards discovering the underlying etiology as to why this disease manifests with a higher incidence in males. The overall incidence of PAES in the general population has been reported to be between 0.17% and 3.5%, with up to 85% of patients diagnosed clinically being males [23–25]. We postulate that confounding variables exist that skew the demographics: the number of cases of PAES in the female population is likely underreported as symptoms may be subclinical or misdiagnosed as CECS, which has a predilection for females [10, 22]. As participation of women in military training and rigorous sports continues to rise, we anticipate seeing an increase in the incidence of PAES in the female population.

## 4. Summary

Diagnosis of popliteal artery entrapment syndrome is fraught with difficulty due to the rarity of the condition and its overlap with other more common diseases. Awareness of key characteristics along with appropriate use of imaging modalities greatly increases the likelihood of making the proper diagnosis. Timely diagnosis is extremely important, both for symptom management and to avoid long-term morbidity. PAES, though predominantly affecting males, should also be considered in young females presenting with a history of exercise-induced claudication.

## Figures and Tables

**Table 1:** Classification scheme of popliteal artery entrapment syndrome.

Popliteal artery entrapment classification	
Type I	Popliteal artery has aberrant medial course around MHG
Type II	Popliteal artery is in normal anatomic position but the MHG inserts more lateral than usual; the artery passes medial and beneath the muscle
Type III	Accessory slip of MHG slings around the artery
Type IV	Artery lies deep in the popliteal fossa entrapped by the popliteus or fibrous band
Type V	Both popliteal artery and vein are entrapped
	MHG: medial head of the gastrocnemius
